# Exploring the elusive composition of *corpora amylacea* of human brain

**DOI:** 10.1038/s41598-018-31766-y

**Published:** 2018-09-10

**Authors:** Elisabet Augé, Jordi Duran, Joan J. Guinovart, Carme Pelegrí, Jordi Vilaplana

**Affiliations:** 1 Secció de Fisiologia, Departament de Bioquímica i Fisiologia, Facultat de Farmàcia i Ciències de l’Alimentació, Universitat de Barcelona, Barcelona, Spain; 2 Institut de Neurociències, Universitat de Barcelona, Barcelona, Spain; 3 Institute for Research in Biomedicine (IRB Barcelona), The Barcelona Institute of Science and Technology, Barcelona, Spain; 4 Centro de Investigación Biomédica en Red de Diabetes y Enfermedades Metabólicas Asociadas (CIBERDEM), Barcelona, Spain; 5 Departament de Bioquímica i Biomedicina Molecular, Universitat de Barcelona, Barcelona, Spain; 6 Centro de Investigación Biomédica en Red de Enfermedades Neurodegenerativas (CIBERNED), Barcelona, Spain

## Abstract

Corpora amylacea (CA) are polyglucosan bodies that accumulate in the human brain during ageing and are also present in large numbers in neurodegenerative conditions. Theories regarding the function of CA are regularly updated as new components are described. In previous work, we revealed the presence of some neo-epitopes in CA and the existence of some natural IgM antibodies directed against these neo-epitopes. We also noted that these neo-epitopes and IgMs were the cause of false staining in CA immunohistochemical studies, and disproved the proposed presence of β-amyloid peptides and tau protein in them. Here we extend the list of components erroneously attributed to CA. We show that, contrary to previous descriptions, CA do not contain GFAP, S100, AQP4, NeuN or class III β-tubulin, and we question the presence of other components. Nonetheless, we observe that CA contains ubiquitin and p62, both of them associated with processes of elimination of waste substances, and also glycogen synthase, an indispensable enzyme for polyglucosan formation. In summary, this study shows that it is imperative to continue reviewing previous studies about CA but, more importantly, it shows that the vision of CA as structures involved in protective or cleaning mechanisms remains the most consistent theory.

## Introduction

Corpora amylacea (CA) are polyglucosan bodies (PGBs) that accumulate primarily in periventricular and subpial regions of the human brain during the ageing process, but are also present in large numbers in selected areas of the brain in several neurodegenerative conditions including Alzheimer’s, Parkinson’s, Huntington’s and Pick’s diseases, as well as in patients with temporal lobe epilepsy^1–9^. Although CA were described by Purkinje as far back as 1837, their origin and function remain unknown.

While essentially constituted of glucose polymers^10^, many components have been attributed to CA. These components are mainly derived from neurons, oligodendrocytes, astrocytes and blood^1,11^. Due to this diversity, many theories have been put forward to explain the origin and functions of CA. Over the years, they have been considered to be protein precipitates of lymphatic or hematogenous origin^12^; the result of a defect in glycogen metabolism^10^; accumulations of breakdown products from neurons and oligodendroglial cells^9^; aggregated remnants of degenerated neuronal cells^13^; conglomerations of interacting proteins from degenerating neurons and extravasated blood elements released after the breakdown of the blood–brain barrier^14^; structures formed from degenerating astrocytes^15^; structures related to a latent human cytomegalovirus infection^16^; and most recently pathological structures related to fungal infections^17^.

CA of human brain share some features with a type of PGB which appear in mouse brain also during the ageing process and frequently named PAS granules because of their positive staining with the periodic acid Schiff (PAS). We recently reported that both CA and PAS granules contain some neo-epitopes that can be recognized by plasmatic natural IgM antibodies^18,19^. Natural antibodies are permanently present in the organisms, even without prior contact with external antigens, and we found that the antibodies directed against the neo-epitopes are present in plasma from mice maintained under specific and opportunistic pathogen-free conditions and also in human umbilical cord sera^18,20^. The results indicated that these organisms are permanently equipped with antibodies prepared to react against the neo-epitopes that arise in CA. We also found that the IgM antibodies that recognize these neo-epitopes are present in the plasma of other mammal species, consistent with the fact that natural antibodies have been fixed by natural selection during evolution and are therefore interspecific^18,20^. Meanwhile, we observed that these natural IgM antibodies are present as contaminants in a high percentage (around 70%) of commercial antibodies obtained from mouse, rabbit, goat and rat, obtained from ascites or serum, being monoclonal or polyclonal, and even supplied as purified^19,20^. Since these contaminant IgMs are recognized by the majority of the anti-IgG antibodies used as secondary antibodies in immunohistochemical studies, they very frequently cause false positive immunostaining in CA. These IgMs therefore account for some of the inconsistencies concerning CA composition and underlie the controversies and the wide variety of theories about their origin and functions.

In this connection, some studies have indicated that CA contain tau protein^21–23^, but others have not reproduced these findings^24^. Contradictory results have also been described with regard to the amyloid-β peptide or its precursor, while some authors reported that it is indeed present in CA^24^, other authors reached the opposite conclusion^23,25^. In a recent study of the presence of both tau protein and amyloid-β peptides using different IgG antibodies directed against these components^18^, our group observed that: a) in all cases in which the vials of primary antibodies were contaminated by IgMs, CA became stained when using a secondary antibody specifically directed against IgM; b) if the vials of primary antibodies were contaminated by IgMs, CA became stained when using non-isotype-specific anti-IgG secondary antibodies; c) pre-adsorption controls prevented the staining of fibrillary tangles and amyloid plaques, but not the staining of CA; and d) with or without IgM contamination, no positive staining of CA was observed when using isotype-specific anti-IgG secondary antibodies. Taken together, these results indicated that the positive staining on CA, when present, was produced by contaminant IgMs but not by the specific IgG antibodies directed against tau protein or amyloid-β peptides; thus, these findings rule out the presence of these components on CA.

Consequently, the presence of other components on CA determined by immunohistochemical procedures and summarized in Table 1 requires clarification. Regarding neuronal markers, positive staining of CA has been observed using NeuN, an antibody directed against neuronal perikarya^25–27^, as well as class III β-tubulin^23^. Components of astrocytic origin have also been described, including specific proteins of the S-100 family^25,26,28,29^, aquaporin 4 (AQP4)^25^ and the glial fibrillary acidic protein (GFAP). In this case, while some authors have reported that the immunoreactivity for GFAP is located throughout the CA^25,26^, other reports specified that CA were outlined by the GFAP signal but that there was no staining within the bodies^24,29–31^. Moreover, CA were reported to contain heat-shock proteins (HSP)^2,26,32,33^ and there is a general consensus on the presence of ubiquitin^2,21,23,25,31^. In the present study, these components will be re-analysed in order to determine which ones are indeed present in CA, taking into account the possible false-positive staining produced by contaminant IgMs.

**Table 1:** Components described on CA by immunohistochemical studies.

Antibody	Epitope	CA staining	Ref.
**Directed against Tau protein**			
Tau-2	phosphatase-independent epitope of tau	+	^22^
Tau 1, clone PC1C6	non-phosphorylated epitope of tau	−	^22^
PHF-1	Tau phosphorylated on ser396 and ser404	+	^21^
TauC3	caspase cleaved tau truncated at asp421	+	^21^
AT8	tau phosphorylated on ser202 and thr205	−	^21^
HT7	full-lenght tau	−	^21^
Tau46	tau C-terminal specific	+	^21^
AT8	tau phosphorylated at ser202 and thr205	−	^23^
Tau-2	human tau	+	^23^
PHF, clone AT100	tau phosphorylated on thr212 and ser214	−	^24^
Tau pS422	tau phosphorylated on ser422	−	^24^
Tau-5	tau 210–241 aminoacids	−	^18^
5E2	tau	−	^18^
**Directed against Amyloid-β (Aβ) or amyloid-β protein precursor (APP)**			
4G8	Aβ 17-24 aminoacids	−	^25^
anti-Aβ 1-42	human Aβ 1-42	−	^23^
anti-Aβ	Aβ 1-40/42	+	^24^
P2	extracytoplasmic APP site	+	^48^
APP-A4, clone 22C11	N-terminal domain of APP	+	^24^
anti-Aβ1–42, 12F4	Aβ 1-42 aminoacids	−	^18^
anti-Aβ1–16, 6E10	Aβ 1-16 aminoacids	−	^18^
**Directed against nestin**			
anti-nestin	nestin	−	^25^
anti-nestin	nestin	+	^26^
**Directed against NeuN**			
anti-NeuN	NeuN protein	+(some)	^25^
anti-NeuN	NeuN protein	+	^26^
anti-NeuN	NeuN protein	+	^27^
**Directed against synuclein (syn)**			
α/β/γ-syn, FL-140	human synuclein	+	^23^
anti-α-syn	α-synuclein protein	+	^24^
anti-α-syn	α-synuclein protein	+(some)	^26^
**Directed against parkin**			
anti-parkin	human parkin	+	^23^
**Directed against reelin**			
G10	reelin N-terminus	+	^24^
clone 142	reelin N-terminus	+	^24^
R12/14	reelin C-terminus	+	^24^
**Directed against microtubule-associated protein 2 (MAP2)**			
anti-MAP2	MAP2	+	^24^
anti-MAP2	MAP2	+	^30^
**Directed against neurofilaments**			
2F11	Neurofilament protein	+(some)	^25^
anti-NF70	Neurofilament protein	−(one+)	^26^
**Directed against S100**			
S100 antisera	S100 proteins (different types, not S100B)	+	^28^
S100B antisera	S100B protein	−	^28^
anti-S100	glial S100 protein	+	^26^
anti-S100	glial S100 protein	+	^25^
anti-S100	glial S100 protein	+	^29^
**Directed against glial fibrillary acidic protein (GFAP)**			
anti- GFAP	GFAP	+(some)	^26^
anti-GFAP	GFAP	+	^25^
anti-GFAP	GFAP	outlining	^24^
anti-GFAP	GFAP	outlining	^31^
anti-GFAP	GFAP	outlining	^30^
anti-GFAP	GFAP	outlining	^29^
**Directed against ubiquitin**			
anti-ubiquitin	ubiquitin	+	^21^
anti-ubiquitin	ubiquitin	+	^25^
anti-ubiquitin	human ubiquitin	+	^31^
anti-ubiquitin	human ubiquitin	+	^23^
anti-ubiquitin	ubiquitin	+	^2^
**Directed against heat-shock proteins (HSP)**			
anti-HSP70	HSP70	+	^26^
anti-HSP27	HSP27	−(some outlining)	^49^
anti-HSP70-72	HSP70-72	−(some outlining)	^49^
anti-HSP90	HSP90	−(some outlining)	^49^
anti-HSP28	HSP28	+	^2^
anti-HSP70	HSP70	+	^2^
anti-HSP72	HSP72	outlining/+	^32^
anti-HSP60	HSP60 383-447	+	^33^
**Directed against aquaporin 4 (AQP4)**			
anti-aquaporin 4	AQP4	+	^25^
anti-aquaporin 4	AQP4	outlining	^31^
**Directed against advanced glycation end-products (AGE)**			
6D12	CML (N-(carboxymethyl)lysine)	+	^42^
anti-pentosidine	pentosidine	+	^42^
**Directed against proteoglycans**			
anti-proteoglycan	keratan sulfate proteoglycan	+	^50^
anti-glycoconjugage	mannose rich glycoconjugate	+	^50^
**Directed against mitochondrial (mit**.**) epitopes**			
anti-Bcl-2	mit. membrane associated protein Bcl-2	+	^51^
**Directed against transcriptional factors**			
anti-c-Jun/AP1	activator prot. 1 transcriptional factor c-Jun	+	^51^
**Directed against transglutaminases (TGs) or products generated by TG**			
anti-TG2	guinea pig TG2	−	^23^
anti-TG1	human TG1	+	^23^
81D4	H-Glu(H-Lys-OH)-OH	+	^23^
**Directed against cytoskeletal proteins**			
anti-β-tubulin III	β-tubulin III	+	^23^
anti-β-actin	β-actin	+	^23^
**Directed against thrombospondin**			
A6.1	thrombospondin1	+	^14^
SPM321	thrombospondin1	+	^14^
**Directed against ADAMTS3**			
anti-ADAMTS13	ADAMTS13	+	^14^
**Directed against hypoxia-inducible factor 1-alpha (HIF-1α)**			
anti-HIF-1α	HIF-1α	+	^25^
**Directed against endothelin B receptor**			
anti-ETBR	endothelin B receptor	+	^52^
**Directed against fungal proteins**			
antifungal antibodies	*C. glabrata, C. famata, C. albicans*, …	+(neurodeg.)	^17^
**Directed against herpesvirus**			
anti-cytomegalovirus	tegument protein pp65	+	^16^

It has also been postulated that CA may be involved in trapping and sequestering potentially hazardous products^1^. Moreover, in view of their extrusion from the marginal glia, it has been proposed that they could be a component of the glio-pial clearance system that removes different molecules from the central nervous system^34^. These theories are based, among other points, on the presence of waste elements in CA as well as the presence of ubiquitin, which is involved in the signaling of substrates for degradation. Moreover, the existence of neo-epitopes on CA and the presence of natural IgM antibodies directed against them reinforce the idea that CA might be structures that are prepared for elimination via the natural immune system, perhaps via phagocytosis processes once extruded from the brain^18^. Accordingly, in the present study we also analyse the presence of certain components that may be related to these functions: specifically, p62, microtubule-associated protein light chain 3 (LC3) and matrix-metalloproteinase-2 (MMP2) proteins. The p62 protein is an ubiquitin-binding scaffold protein that collects ubiquitinated proteins via their C-terminal Ubiquitin-Associated (UBA) domain, and recent studies suggest that the binding of ligands to the p62 ZZ domain induces p62 self-oligomerization and aggregation^35^. As p62 also binds directly to LC3 and GABARAP family proteins, it can therefore escort the ubiquitinated proteins to the autophagosomes, which can then be directed to autophagy processes^36^ or to secretory autophagy, in which the content of the autophagosome becomes extruded from the cell^37^. The MMP2 protein is a metalloproteinase protein involved in the extracellular remodelling^38^, a process necessary for the extrusion of CA from marginal astrocytes to the cerebrospinal fluid. We also analysed the presence of glucose synthase (GS) on CA. GS is the only enzyme able to synthesize glucose polymers in mammals and, as indicated above, glucose polymers are the most abundant component of CA. Remarkably, genetic ablation of glycogen synthase in mouse brain avoids the formation of PGBs in this organ^39^. Although the presence of GS on CA has not been studied to date, its presence has been observed in other PGBs such as both neuronal Lafora bodies and CA-like granules in malin^KO^ mice, a mouse model of Lafora disease^40,41^.

Accordingly, in order to shed light on CA composition and function, the present study has two main objectives: 1) to verify the presence on CA of some previously described components, and 2) to determine the presence of certain potential components that have not been studied to date.

## Results

### Revision of the presence of some components previously described in CA

**Figure 1:**
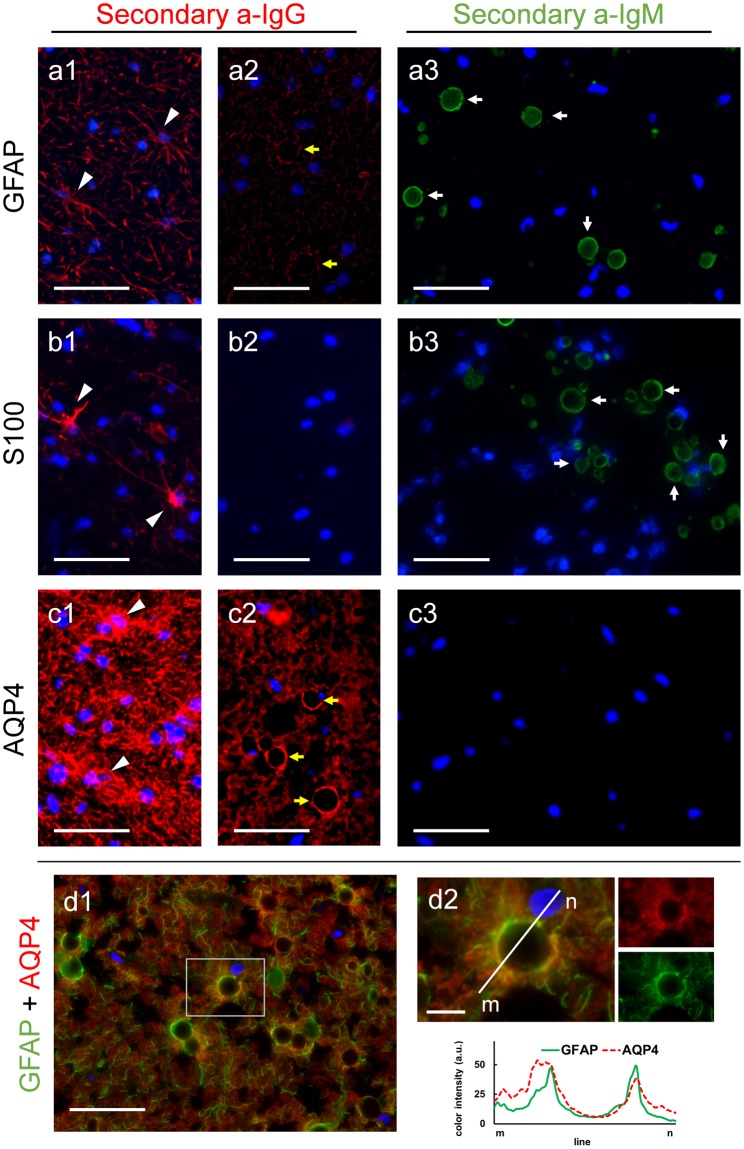
Absence of GFAP, S100 and AQP4 in CA. Representative images of human brain hippocampus sections immunostained with GFAP (**a**), S100 (**b**) and AQP4 (**c**). When using isotype-specific anti-IgG antibodies (**a1**, **a2**, **b1** and **b2**) or anti-IgG(H&L) antibodies (**c1**–**c2**) conjugated to a red fluorochrome in the second incubation, the positive labeling of astrocytes can be observed (arrowheads in **a1**, **b1** and **c1**), but CA are not labelled and are visible just as a dark hole when astrocyte processes encircled them (yellow arrows in **a2** and **c2**). By using an isotype-specific anti-IgM antibody conjugated to a green fluorochrome in the second incubation, labeling of CA can be observed as shown in **a3** and **b3** (white arrows) but not in **c3**, indicating that GFAP and S100, but not AQP4, are IgM contaminated. Double immunostaining with GFAP and AQP4 show these two markers surrounding the CA (**d1**). **d2** shows an inset of **d1**, where a CA is magnified. The different color channels are shown in small images next to the corresponding ones, and the histogram profiles of green and red intensities on the plotted line show the similar distribution of both markers around the CA. Hoechst (blue) was used for nuclear staining. Scale bar in **d2**: 10 μm, other scale bars: 50 μm.

Immunohistochemical studies were performed in order to verify the presence of some of the proposed components in CA. Prior to these studies, we used PAS staining to confirm the presence and determine the localization of CA on the hippocampus of each donor (medical data from donors are specified in the methods section). As expected, all the brains contained CA, which were found mainly lining the periventricular and subpial regions (Supplementary Figure S1). Moreover, it must be taken into account that in previous studies with samples from AD and non-AD patients, we did not observe morphological or staining differences on CA depending on the presence or the absence of AD pathology^18^ and so did happen in the present work, in which sections from a minimum of one non-AD and three AD brain donors were used for each specific staining. Representative staining of AD and non-AD can also be observed in supplementary Figure S1. Thereafter, we considered the following results as general observations for all CA, and all representative images presented in the article are obtained from brain sections of AD patients. Some of the proposed components in CA are the astrocytic GFAP and S100B proteins. For these markers we tested the staining on different hippocampal sections from each patient, using the isotype-specific anti-IgG secondary antibody and also a specific anti-IgM secondary antibody. A positive staining with the specific anti-IgG secondary antibody would indicate the presence of the specific marker, while positive staining with the secondary anti-IgM antibody would indicate the presence of contaminant IgMs directed against the neo-epitopes of CA in the vial of the primary antibody. Figure 1 shows representative images from the different staining. When using the appropriate isotype-specific anti-IgG secondary antibodies, the astrocytic processes became stained with the GFAP or S100B primary antibodies, and in some cases the whole astrocyte could be visualized (Fig. 1a1 and b1). These observations become the positive control of the staining, because they exhibited the expected staining of astrocytes with the GFAP and S100 markers. However, none of these markers were present in CA (Fig. 1a2 and b2). In the case of GFAP staining, some CA appeared as unstained black spheres surrounded by astrocytic processes (Fig. 1a2). When the staining was performed with the respective anti-IgMs, in both cases CA became stained (Fig. 1a3 and b3). These results indicated the presence of contaminant IgMs in the vials of primary antibodies, which recognize the neo-epitopes of CA; they also indicated that if a non-isotype-specific anti-IgG secondary antibody had been used, such as an anti-IgG against heavy and light chains (anti-IgG(H&L)), CA would have become stained due to the cross-reaction of this secondary antibody with the contaminant IgMs. We also tested the presence of AQP4 in CA. In this case, although the commercial isotype-specific anti-IgG secondary antibody was not available, the presence of this marker in CA was ruled out because there was no positive labeling of CA either with the anti-IgG(H&L) or with the anti-IgM antibody (Fig. 1c1-c3). If contaminant IgMs had been present, a positive staining with anti-IgG(H&L) antibody would have been observed in CA. This hypothetical case would have led to a controversial result because of the possible cross-reaction of this secondary antibody with the IgMs. It should be pointed out that when using anti-IgG(H&L) there was no staining in CA, but there was positive labeling of astrocytes (Fig. 1c1), confirming the correct functionality of the primary antibody. As in the case of the GFAP staining, AQP4 signals appeared surrounding CA, leading to empty spheres. The double staining with GFAP and AQP4 showed that these two markers are mainly located surrounding the CA (Fig. 1d1 and d2), and the color histogram profiles showed a similar distribution of both markers around the CA. Taken together, these results indicate that CA do not contain either GFAP, S100B or AQP4, and the presence of these astrocytic markers reported elsewhere were probably due to false staining caused by the contaminant IgMs.

**Figure 2:**
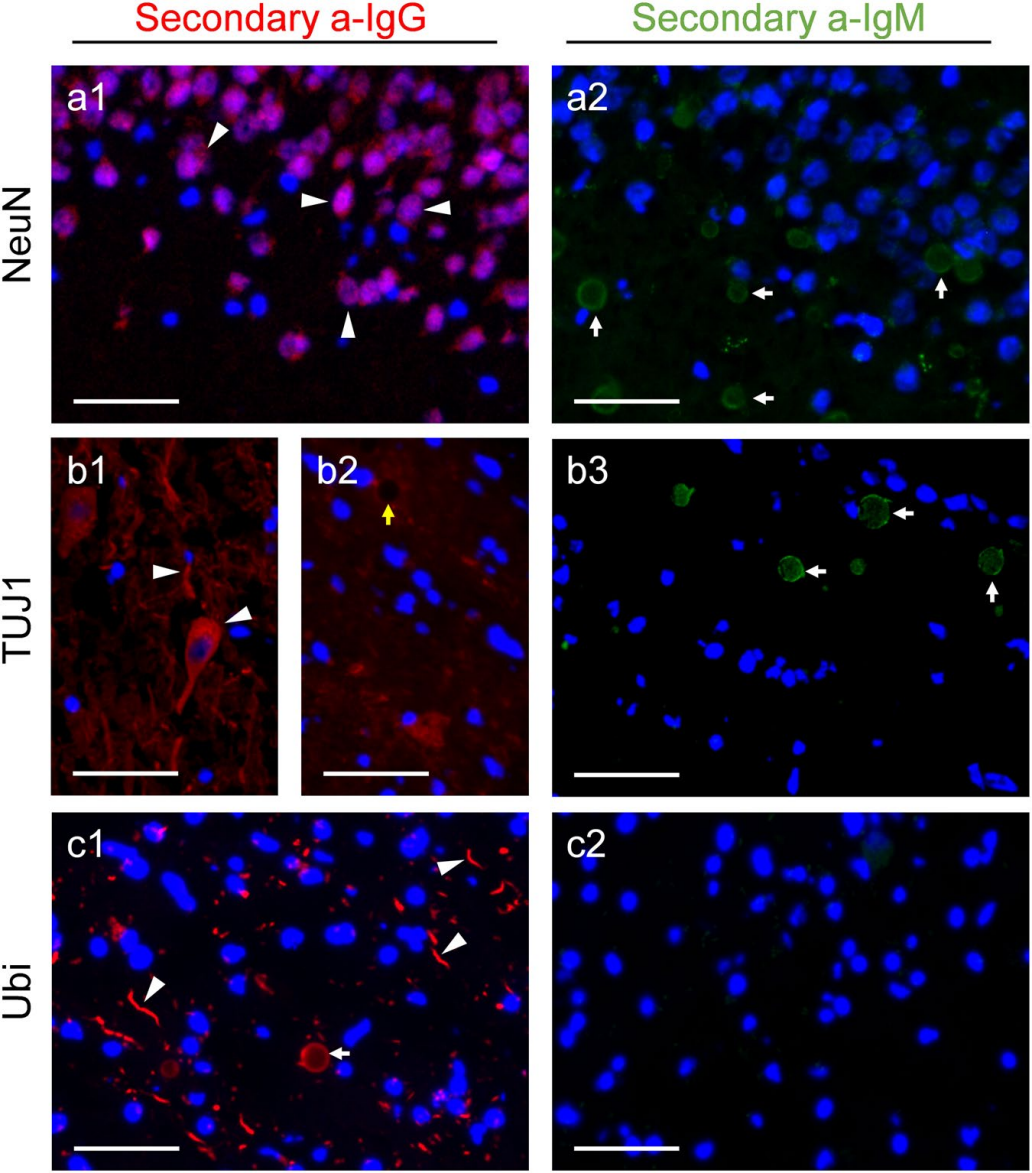
Absence of NeuN and class III β-tubulin and presence of ubiquitin in CA. Representative images of human brain hippocampus sections immunostained with NeuN (**a**), anti-class III β-tubulin (TUJ1) (**b**) and anti-ubiquitin (Ubi) (**c**). As expected, when using isotype-specific anti-IgG antibodies conjugated to a red fluorochrome in the second incubation, NeuN stained the neuronal soma (arrowheads in **a1**), TUJ1 stains both neuronal soma and neurites (arrowheads in **b1**), and Ubi stained some dystrophic neurites (arrowheads in **c1**). CA were not labeled with NeuN (**a1**) and TUJ1 (yellow arrow in **b2**), but became labeled with Ubi (white arrow in **c1**). When using isotype-specific anti-IgM antibodies conjugated to a green fluorochrome in the second incubation, labeling of CA was observed in **a2** and **b3** (white arrows), but not in **c2**, indicating the presence of contaminant IgMs in the vials of NeuN and TUJ1, but not in the Ubi vial. Hoechst (blue) was used for nuclear staining. Scale bar: 50 μm.

We also verified the presence of the neuronal markers NeuN and class III β-tubulin on CA. When using isotype-specific anti-IgG secondary antibodies, the presence of these components is observed in the expected structures, i.e., the neuronal perikarya in the case of NeuN (Fig. 2a1) and certain neurons and neurites in the case of TUJ1, which recognises class III β-tubulin (Fig. 2b1). However, neither NeuN nor TUJ1 labeled CA (Fig. 2a1 and b2). When simple immunofluorescences were performed using the anti-IgMs as secondary antibodies, in both cases CA appeared labeled, confirming the presence of contaminant natural IgMs on the vials of the primary antibodies (Fig. 2a2 and b3). Again, these IgMs would have produced false-positive staining on CA, if a non-isotype-specific anti-IgG antibody had been used. All things considered, NeuN and TUJ1 do not label CA, and the previous descriptions of NeuN and class III β-tubulin are also probably based on false-positive staining produced by contaminant IgMs.

We also rechecked the presence of some components associated with the possible role of CA, including the ubiquitin protein and the HSP70. In the case of ubiquitin, using an anti-ubiquitin (Ubi) as primary antibody, CA and some dystrophic neurites became labeled with the isotype-specific anti-IgG secondary antibody (Fig. 2c1), but not with the anti-IgM secondary antibody (Fig. 2c2). Thus, the presence of ubiquitin on CA was confirmed. In the case of HSP70, CA was not labeled when using the isotype-specific anti-IgG secondary antibody (data not shown). However, in this case we did not observe any positive region or structure that could be used as a positive control, and so we could not rule out the possibility of a methodological problem with the primary antibody or epitope preservation. Consequently, the presence of HSP70 on CA remains doubtful. In any case, we observed that the CA became stained when using the anti-IgM secondary antibody, indicating that the vial of the primary antibody is also contaminated by IgM. Consequently, if a non-isotype-specific anti-IgG had been used, the CA labeling might have been positive, but due to a false-positive staining of CA.

### Study of the presence of certain components predicted in CA

As indicated in the introduction section, and because CA could be involved in the sequestration of potentially hazardous products and their brain extrusion, we studied here the presence of p62, LC3 and MMP2 in CA. The presence of GS, the only enzyme able to synthesize glucose polymers, was also evaluated.

**Figure 3:**
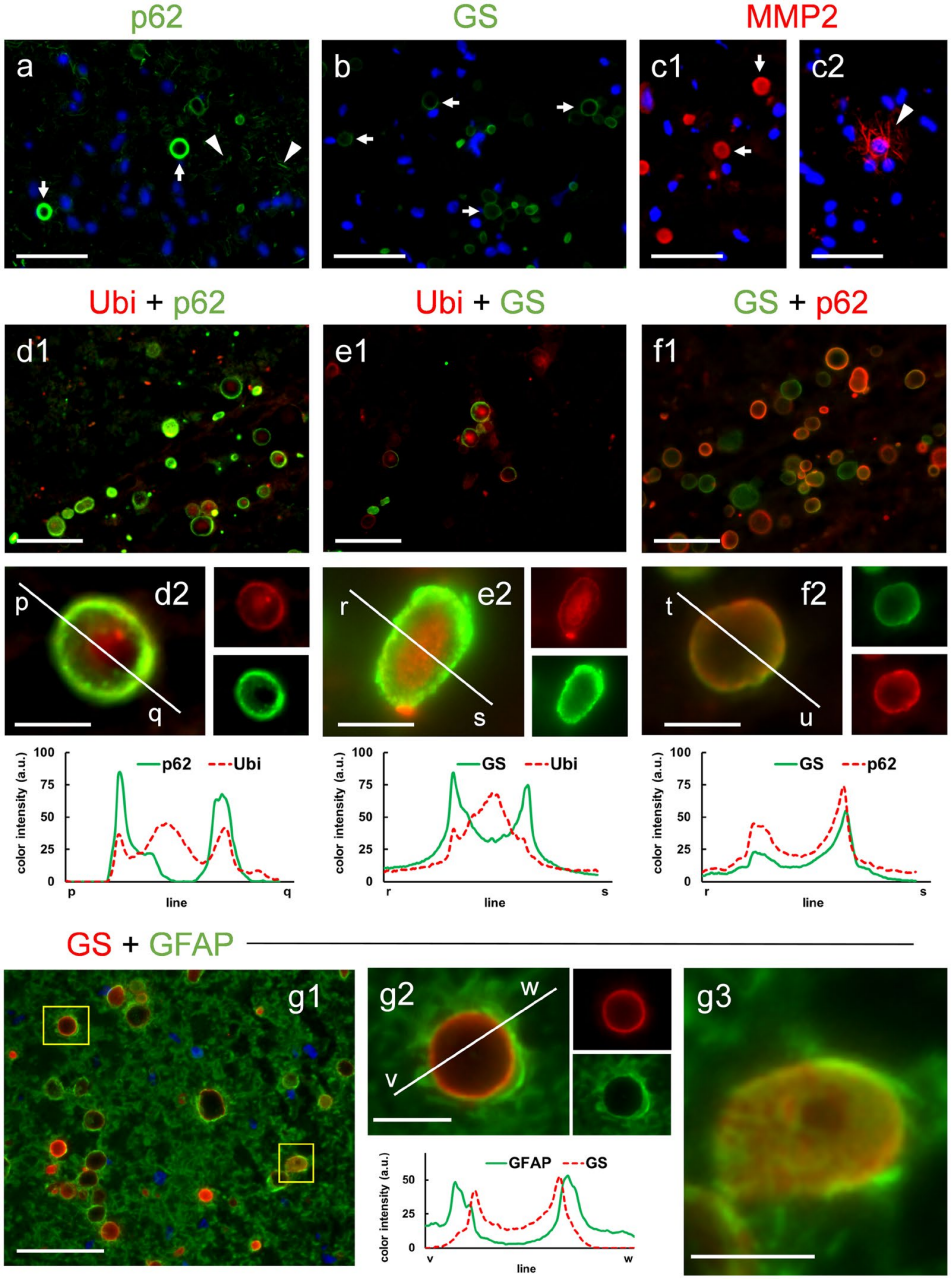
Immunolabeling of CA with the markers p62, GS and MMP2. Representative images of human brain hippocampus sections immunostained with p62 (**a**), GS (**b**) and MMP2 (**c1** and **c2**). The three markers positively stained the CA (arrows). p62 also stained some dystrophic neurites (arrowheads in **a**), while MMP2 stained some thin filaments (arrowheads in **c2**). Hoechst (blue) was used for nuclear staining. **d1**–**d2**: double immunostaining with anti-ubiquitin (Ubi) (red) and p62 (green); **e1**–**e2**: double immunostaining with Ubi (red) and GS (green); **f1**–**f2**: double immunostaining with GS (green) and p62 (red). In some CA, p62 and GS are clearly visible in the peripheral region (**d2**, **e2** and **f2**), while ubiquitin is concentrated in the central zone (**d2** and **e2**). The different color channels from **d2**, **e2** and **f2** are shown in small images next to the corresponding ones. The histogram profiles of green and red intensities on the plotted lines illustrate the peripheral location of p62 and GS, and show that ubiquitin is concentrated in the central zone but is also present at the periphery. Double staining with GS and GFAP showed that GFAP staining is surrounding that of GS, which indicated that the astrocytic processes are surrounding CA (**g1**). This is clearly shown when CA is magnified (**g2**). When the histogram profiles are traced, it can be observed that the peaks of intensity of GFAP staining appear externally to those of GS staining. In some cases, CA become sliced in a tangential plane, and then the GS staining appear throughout the surface of the CA, and the astrocytic processes stained with GFAP are surrounding this surface (**g3**). Scale bars on **d2**, **e2**, **f2**, **g2** and **g3**: 10 μm; other scale bars: 50 μm.

First, we checked the presence of contaminant natural IgMs in each of these four commercial antibodies by performing the immunohistochemistry with the appropriate anti-IgM secondary antibodies. With this approach, the only case in which the CA became stained was when LC3B was used (data not shown). Thereafter, the only primary antibody that contained IgMs was the LC3B. As LC3B is a polyclonal antibody obtained in rabbit, and the available anti-rabbit IgG antibodies are not isotype-specific, these anti-IgG antibodies could crossreact with the IgMs. Therefore, in this case we were not able to determine whether or not LC3B was present in the CA. In the other cases, i.e., the antibodies directed against p62, GS and MMP2, which are free of contaminant IgMs, we observed a positive staining of CA by using the respective anti-IgG secondary antibodies (Fig. 3a–c). We therefore concluded that the three components (p62, GS and MMP2) were present in CA. On the other hand, taking into account that p62 and ubiquitin may interact due to the UBA domain present in p62, we also studied the co-localization pattern of these two proteins in CA and the co-localization of GS and p62 and that of GS and ubiquitin. After performing double staining with Ubi and p62, we observed different staining patterns in CA. Most of them presented p62 and ubiquitin in the peripheral area, generating a ring or a circle, and can contain ubiquitin, but no p62, concentrated in the central part (Fig. 3d1 and d2). In some cases (Fig. 3d1) CA presented p62 staining uniformly throughout the body, which is produced by the section in a tangential plane on the surface of the CA. The double staining with Ubi and GS presented similar patterns to those previously described. GS was located mainly on the periphery of the CA, where it might be accompanied by ubiquitin, and in the center of the CA there was in some occasions a region with high content of ubiquitin (Fig. 3e1 and e2). Like the previous staining, the double staining with GS and p62 showed that these two components are mainly located at the periphery of the CA, with GS staining predominating in some cases and p62 staining in others (Fig. 3f1 and f2). Neither of these two last components appeared concentrated in the central part of the CA. The histogram profiles shown in Fig. 3 indicate that the ubiquitin pattern in CA differs from those of GS and p62. GS and p62 are located mainly in the periphery of the structure, while ubiquitin is located in the peripheral region but may also appear concentrated in the center. In order to corroborate these points, we obtained the 3D reconstruction of some representative CA stained with the different markers. As can be observed in supplementary videos V1–V4, and as indicated before, GS, p62 and Ubi stain the periphery of CA, while Ubi staining can also be concentrated in the central region of the structure. The double staining with GS and GFAP showed that GFAP staining is surrounding that of GS, which indicated that the astrocytic processes are surrounding CA (Fig. 3g1). This is clearly shown when CA is magnified (Fig. 3g2). When the histogram profiles are traced, it can be observed that the peaks of intensity of GFAP staining appear externally to those of GS staining. In some cases, CA become sliced in a tangential plane, and then the GS staining appear throughout the surface of the CA, and the astrocytic processes stained with GFAP are covering this surface (Fig. 3g3).

The results of the immunostaining performed in this study, including both the rechecking of the components described in CA and the study of the presence of some previously unreported components, are summarized in Table 2.

**Table 2:** Summary of the results obtained with the antibodies used for immunohistochemistry.

		CA staining			Presence on CA^b^	Positive controls	
		secondary antibody				secondary antibody	
		α-IgG isotype-specific	α-IgG non-isotype-specific^a^	α-IgM		α-IgG isotype-specific	α-IgG non-isotype-specific
primary antibody (IgG)	GFAP	−		+	negative	astrocytes	
	S100	−		+	negative	astrocytes	
	AQP4		−	−	negative		astrocytes
	NeuN	−		+	negative	neuronal soma	
	TUJ1	−		+	negative	neuronal mt.	
	HSP70	−		+	doubtful	not observed	
	Ubi	+		−	positive	dys. neurites	
	p62	+		−	positive	dys. neurites	
	GS		+	−	positive		polyglucosans
	MMP2		+	−	positive		thin filaments
	LC3B		+	+	doubtful		not observed

^a^α-IgG non-isotype-specific secondary antibodies were used when the isotype-specific antibodies were unavailable.

^b^Presence on CA can be assumed only when the antibody labels the expected structure (positive control) and (a) the secondary antibody is isotype-specific, or (b) the secondary is non-isotype-specific but the vial of the primary antibody is not IgM contaminated. mt.: microtubules; dys.: dystrophic.

## Discussion

The interpretation of the function of *corpora amylacea* has varied over time, and in fact numerous theories about these bodies have been formulated, due mainly to the continuous description of new components. In previous work, we were able to rule out the presence of some of these components, specifically the β-amyloid peptides and the tau protein^18^. In the present study we have expanded the list of components erroneously attributed to CA. Contrary to the previous descriptions summarized in Table 1, we have seen that CA do not contain any of the following astrocytic or neuronal markers: GFAP, S100, AQP4, NeuN and class III β-tubulin. Except for the antibody against AQP4, all the antibodies tested contained contaminant IgMs that would have given false positives if non-isotype-specific secondary antibodies had been used. Thus, it is not surprising that conflicting results regarding the presence of these markers in CA have been reported, and the assumed presence of these components^25–27^ cannot be used to define the neuronal or astrocytic origin of CA. According to the descriptions in Table 1, there remains some other compounds whose presence should be verified in CA, notably the ones that present discrepancies, such as nestin, α-sinuclein or the transglutaminases, but also the components that do not present discrepancies at present but have been described only once, such as thrombospondin1, ADAMTS13, reelin, some markers related to cytomegalovirus infections or some others related to fungal components. Consequently, until the presence of these components on CA has been confirmed, caution should be taken regarding the theories about the origin of CA that are based on the presence of these components. One theory, based on the presence of thrombospondin1 and ADAMTS13, considers CA as conglomerations of interacting proteins from degenerating neurons and extravasated blood elements^14^. Another theory proposes that CA consist of accumulations of breakdown products from neurons and oligodendroglial cells^9^, and is based on the presence of amyloid and tau proteins (discarded in our previous study^18^), together with a significant proportion of substances derived from oligodendrocytes and/or myelin and detected via immunohistochemistry. Other authors suggest that changes in reelin levels play a role in the formation of CA by altering cytoskeletal dynamics^24^; however, that study was based on the positive staining with antibodies directed against β-amyloid peptides and tau protein (discarded in our previous study^18^) and α-synuclein and reelin, which must be revised. Other theories based on immunohistochemistry procedures that also need to be verified associate CA structures with a latent human cytomegalovirus infection^16^ or consider CA as pathological structures related to fungal infections or even originated from mycoses^17^. Elucidating which components really belong to CA will help to focus the theories about CA functionality.

Remarkably, interpretations of CA often omit the fact that these bodies are mainly formed by aggregates of glucose polymers, and the presence of minority components, which are often erroneously identified, may have been overestimated and overemphasized. Some authors postulate that PAS staining of CA is caused by the non-enzymatic glycation of oxidized proteins and lipids forming Schiff bases and Amadori products^42^. However, although we cannot discard the presence of glycated substances, the following must be taken into account: a) isolated CA contain almost 70% of hexoses; b) enzymatic studies indicate that the polyglucosan structure of CA contain both α-1:4 and α-1:6 glucosidic linkages; c) there is no evidence that CA contain lipids or nucleic acids; and d) CA are also stained with PAS-dimedone (which does not stain acid mucopolysaccharides, glycoprotein, or mucoprotein) but not with the ninhydrin-Schiff technique (thereby indicating absence or low amount of protein)^10^. Consequently, the glycation of oxidized proteins and lipids is not sufficient to explain the high content and the nature of the glucose polymers present in CA. Moreover, we recently detected that malin^KO^ mice, a mouse model of Lafora disease, have two types of polyglucosan bodies in their brain, i.e. neuronal Lafora bodies and astrocytic PAS granules^41^. These astrocytic PAS granules, which are also present in aged mouse from other strains^43^, are analogous to human CA and defined as CA-like (CAL) granules^41^. The ablation of GS in these malin^KO^ mice (i.e. double mutant GS^KO^ malin^KO^) prevents the formation of both types of granules^44^ and aged GS^KO^ mice do not present CAL granules^41^. These two observations indicate that GS is indispensable for the formation of these granules and that the non-enzymatic glycation of oxidized proteins is not sufficient to generate them.

In the present study, we identified ubiquitin, p62 and GS as components of CA. The presence of GS is not surprising if we take into account that both the CA and the other PGBs are structures that can reach tens of microns in diameter, requiring large amounts of polyglucosans to be formed. Our results show that the staining of GS is mainly located in the peripheral area of CA. In some cases, the peripheral immunostaing of a specific globular structure is an artifact produced by problems of antibody penetration. However, this cannot be the present situation: CA attain up to 30 µm of diameter and, as brain sections in our study are 6 µm-thick, most of them must have been sliced and their central part exposed. In fact, sliced CA can be observed in the 3D animations shown in supplementary videos. Thus, the peripheral staining of GS indicates that a) GS is mainly located in the peripheral area of CA or b) GS located inside have suffered some physical or chemical modification that prevents its detection via immunohistochemistry. For its part, p62 stain was also mainly present in the peripheral area. As this protein has been related with the collection of ubiquitinated proteins^35^, we expected some colocalization between p62 and ubiquitin. Indeed, we found a certain amount of ubiquitin in the periphery of the CA, but in some cases ubiquitin also appeared concentrated in the central part of CA, where p62, as GS, is absent or perhaps is physically or chemically altered.

Accordingly, all these findings indicate that CA are not just the aggregation of different insoluble glucose polymers amassing some proteins like GS, p62 and ubiquitin, but polyglucosan structures with two differentiated regions: the outer part, in which predominates the GS and p62 but also contain ubiquitin, and the central part, in which high amount of ubiquitin, but not GS or p62, can be immunohistochemically detected. It can be speculated that the outer part of CA is a dynamic or growing region, where GS are forming the glucose polymers and where p62 might capture the ubiquitinated substances, and that the central part is a stable region where the polyglucosans become accumulated and retain the ubiquitinated substances. This conjecture is consistent with the recent hypothesis indicating that corpora amylacea are waste containers in which deleterious or residual products are isolated^18^. An alternative explanation is that the glucose polymers themselves are the waste substance. In fact, the polyglucosans of all PGBs present alterations in the frequency of branching, in the phosphate levels and water solubility with respect to physiologically functional glycogen^1^. However, if glucose polymers are the waste substance in CA, it must be determined the nature of the ubiquitinated substances that become accumulated, and it is necessary to explain why p62 and GS are found mainly in the peripheral region.

In addition to glucose polymers in CA, the presence of waste substances has also been described by numerous studies, some of which were not based on immunostainings. In this regard, some authors used a combination of two-dimensional electrophoresis with matrix-assisted laser desorption/ionization-time of flight mass spectrometry and database interrogations to perform a proteomic analysis of CA from multiple sclerosis brain lesions collected by laser microdissection. These authors identified several proteins of suspected neuronal origin and others involved in the regulation of apoptosis and senescence. Those findings thus supported the notion that the biogenesis of CA involves the degeneration and aggregation of substances of neuronal origin^13^. Ultrastructural studies of vestibular nerve fragments surgically removed from patients with Meniere’s disease and vascular cross-compression syndromes suggest that CA, originated in astrocytes, accumulate abnormal material and are extruded from the marginal glia out of the nervous system^34^. Combining results of human neuropathological surveys, cell culture techniques and animal models, some authors propose that CA are homologous to ‘Gomori-positive’ granules that accumulate in subcortical/periventricular regions of the rodent brain^45^. These granules are derived from damaged mitochondria engaged in a complex macroautophagic process. Accordingly these authors proposed a “mitophagy” hypothesis to explain CA formation^46^. A more recent article^47^ suggests that the biogenesis of CA can be envisioned in the context of a revised free radical-mitochondrial-metal theory of CNS ageing, in which sustained up-regulation of the stress protein heme oxygenase-1 in astrocytes may play a pivotal role. Up-regulation of heme oxygenase-1 can lead to the release of intracellular iron and carbon monoxide, thereby promoting the transformation of normal mitochondria into Gomori granules and CA^8^. It must be pointed out that although immunohistochemical evidence in those studies may be subject to the concerns raised in the present work, mitochondrial remnants within CA have been widely demonstrated in ultrastructural studies.

All these theories on CA, including that of neuronal origin, astrocytic origin and Gomori-astrocytic origin, overlook the presence of a high content of carbohydrates in these bodies and are incongruous. However, they all share the description of the presence of abnormal materials or waste elements, and may be consistent with the notion of CA being containers in which waste products are isolated for later removal by the natural immune system^18^. In this context, conditions like ageing, neurodegenerative diseases and the toxically induced production of reactive oxygen species, all of which trigger the formation of abnormal materials, would result in an increase in CA. In a recent study using electronic microscopy techniques we observed that some CA have a central highly dense region compatible with the dense region observed in the present study with a high content of ubiquitin. Moreover, we also determined that the neo-epitopes recognized by natural IgMs are distributed throughout the CA, including the central part. It is therefore consistent that we observed these neo-epitopes (which can be markers of substances to be eliminated by the natural immune system) in the same areas in which ubiquitin (a marker of waste substances) were detected.

In summary this study shows that it is imperative to continue reviewing previous studies about CA. But, more importantly, it shows that the vision of CA as structures involved in protective or cleaning mechanisms remains the most consistent theory.

## Methods

### Human brain samples

Post-mortem brain samples, i.e. frozen hippocampal sections with a thickness of 6 µm, stored at −80 °C, were obtained from the *Banc de Teixits Neurològics* (Biobanc-Hospital Clínic-IDIBAPS, Barcelona). Sections were taken from four patients with neuropathologically confirmed Alzheimer’s disease (A3B3C3 stage, 81–88 years old) and one non-AD patient with subcortical ischaemic vascular dementia (70 years old). The medical data of these patients are detailed in Table 3.

**Table 3:** Medical data about brain donors.

Subject	Gender	Age of death (years)	Post-mortem delay (hh:mm)	Neuropathological diagnosis^a^	Braak stage – CERAD	Others^b^
1	Female	81	4:30	AD	V - C	CAA
2	Female	85	5:00	AD	V - C	CAA
3	Female	87	5:45	AD	VI - C	CAA
4	Male	88	3:00	AD	V - C	CAA
5	Male	70	4:30	SIVD	none	IFCM

^a^AD: Alzheimer’s disease with high level of Alzheimer’s disease neuropathological change (A3, B3, C3); SIVD: subcortical ischaemic vascular dementia (small vessel disease, basal ganglia and pontine lacunar infarcts).

^b^CAA: cerebral amyloid angiopathy; IFCM: isolated frontal cortex microinfarct.

All procedures involving human samples were performed in accordance with appropriate guidelines and regulations. All experiments involving human tissue were approved by the Bioethical Committee of the University of Barcelona (IRB00003099).

### PAS staining

Frozen hippocampal sections were stained with PAS following the standard procedure described previously^18^. Briefly, sections were fixed for 10 min in Carnoy’s solution (60% ethanol, 30% chloroform and 10% glacial acetic acid). The slides were then pretreated for 10 min with 0.25% periodic acid (19324-50, Electron Microscopy Sciences) in distilled water, followed by a washing step for 3 min with distilled water. They were then immersed in Schiff’s reagent (26052–06, Electron Microscopy Sciences) for 10 min. Next, the slides were washed for 5 min in distilled water. Nuclei were counterstained for 1 min with a hematoxylin solution following Mayer (3870, J.T. Baker, Center Valley, USA). They were then washed, dehydrated with xylene, and coverslipped with quick-mounting medium (Eukitt, Fluka Analytical, Germany).

### Antibodies and reagents for the immunohistochemistry

The following antibodies were used as primary antibodies: mouse monoclonal IgG1 against NeuN (clone A60; 1:200; MAB377; MerckMillipore, Darmstadt, Germany), mouse monoclonal IgG1 against GFAP (clone GA5; 1:800; MAB360; MerckMillipore), mouse monoclonal IgG2a against class III beta-tubulin (clone TUJ1; 1:50; 801201; Biolegend, San Diego, CA, USA), mouse monoclonal IgG1 against S100 β-subunit (clone SH-B1; 1:500; S2532; Sigma-Aldrich), mouse monoclonal IgG2a against p62 (1:200; ab56416; Abcam, Cambridge, UK), rabbit monoclonal IgG against GS (1:100; 15B1; Cell Signaling, Leiden, Netherlands), mouse monoclonal IgG1 against ubiquitin (clone Ubi-1; 1:200; ab7254; Abcam), mouse monoclonal IgG1 against HSP70 (clone C92F3A-5; 1:100; MA1-10889; Thermo Fisher Scientific, Rockford, IL, USA), rabbit polyclonal IgG against AQP4 (1:100; AB3594; MerckMillipore), rabbit polyclonal IgG against MMP2 (1:200; AB19167; MerckMillipore) and rabbit polyclonal IgG against LC3 (LC3B; 1:200; NB100-2220; Novus Biologicals, Abingdon, UK).

The following antibodies were used as secondary antibodies: goat anti-rabbit IgM µ chain conjugated to fluorescein isothiocyanate (FITC) (1:250; ab98458; Abcam), Alexa Fluor (AF) 488 goat anti-mouse IgM µ chain specific (1:250; 115-545-075; Jackson ImmunoResearch Laboratories, Newmarket, UK), AF594 goat anti-mouse IgG1 (1:250; A-21125; Life Technologies); AF488 goat anti-mouse IgG2a (1:250; A-21131; Life Technologies), AF594 goat anti-mouse IgG2a (1:250; A-21135; Life Technologies), AF488 goat anti-mouse IgG1 (1:250; A-21121; Life Technologies), AF555 donkey anti-rabbit IgG directed against both their heavy and their light chains (1:250; A-31572; Life Technologies) and AF488 donkey anti-rabbit IgG directed against both their heavy and their light chains (1:250; A-21206; Life Technologies).

### Immunohistochemistry

For the immunohistochemistry, the frozen hippocampal sections (6 µm thick), stored at −80 °C, were air dried for 10 min and then fixed with acetone for 10 min at 4 °C. After 2 h of drying, sections were rehydrated with phosphate-buffered saline (PBS) and then blocked and permeabilized with 1% bovine serum albumin in PBS (Sigma-Aldrich) (Blocking buffer, BB) with 0.1% Triton X-100 (Sigma-Aldrich) for 20 min. They were then washed with PBS and the primary incubation was overnight at 4 °C. Next, the slides were washed and incubated for 1 h at room temperature with the secondary antibody. Nuclear staining was performed with Hoechst (2 μg/mL, H-33258, Fluka, Madrid, Spain) and the slides were washed and coverslipped with Fluoromount (Electron Microscopy Sciences, Hatfield, PA, USA). Staining controls were performed by incubating the secondary antibody after the incubation with BB instead of the primary antibody. In double stainings, controls for cross reactivity of the antibodies were also performed.

### Image acquisition and processing

Images were captured with a fluorescence laser and optical microscope (BX41, Olympus, Germany) and stored in TIFF format. All the images were acquired using the same microscope, laser and software settings. Exposure time was adapted to each staining, but the respective control images were acquired with the same exposure time. Image analysis and treatment were performed using the ImageJ program (National Institute of Health, USA). Images that were modified for contrast and brightness to enhance their visualization were processed in the same way as the images corresponding to their respective controls. In some images from double staining, the histogram profile of the intensity of the different colors along one drawn line was obtained by the ImageJ program.

A Hamamatsu NanoZoomer Digital Slide Scanner (40X magnification) was used to obtain low power microphotographs of brain sections stained with the PAS technique.

In order to study the distribution of some markers on CA, image stacks were taken with a confocal laser scanning microscopes (LSM 880, Zeiss, Germany) and 3D animations were obtained by means of the Image J program (National Institute of Health, USA).

## Electronic supplementary material

Video V1

Video V2

Video V3

Video V4

Figure S1
