# Supplementary figures and images for: Exploring the elusive composition of corpora amylacea of human brain
Source: Sci Rep. 2018 Sep 10;8:13525. doi: 10.1038/s41598-018-31766-y (PMC6131176; doi:10.1038/s41598-018-31766-y)

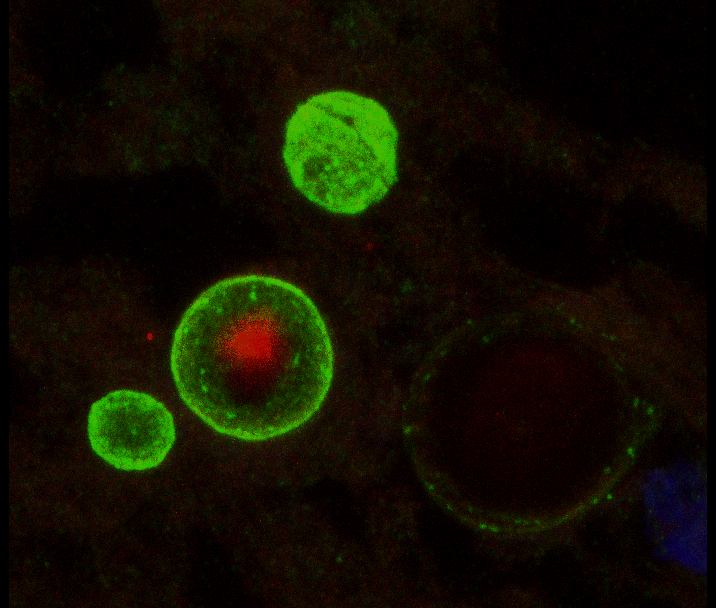

Supplement: Supplementary file 1 — Video V1 [file 41598_2018_31766_MOESM1_ESM.gif]

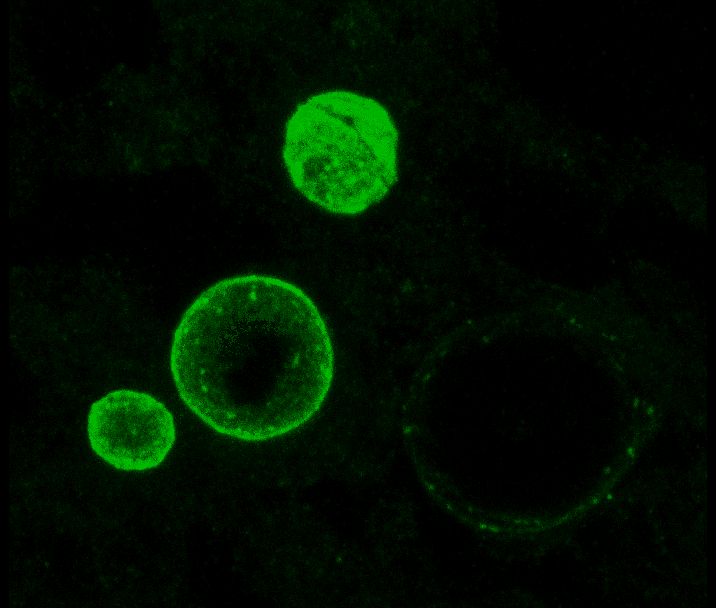

Supplement: Supplementary file 2 — Video V2 [file 41598_2018_31766_MOESM2_ESM.gif]

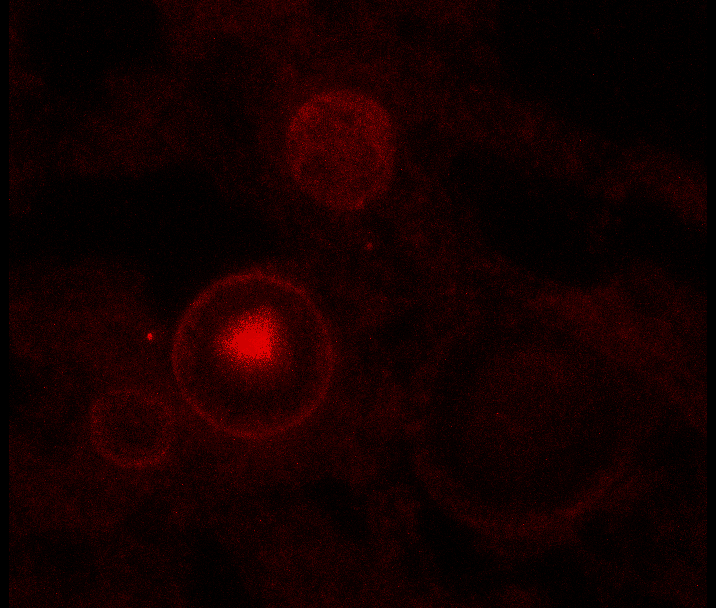

Supplement: Supplementary file 3 — Video V3 [file 41598_2018_31766_MOESM3_ESM.gif]

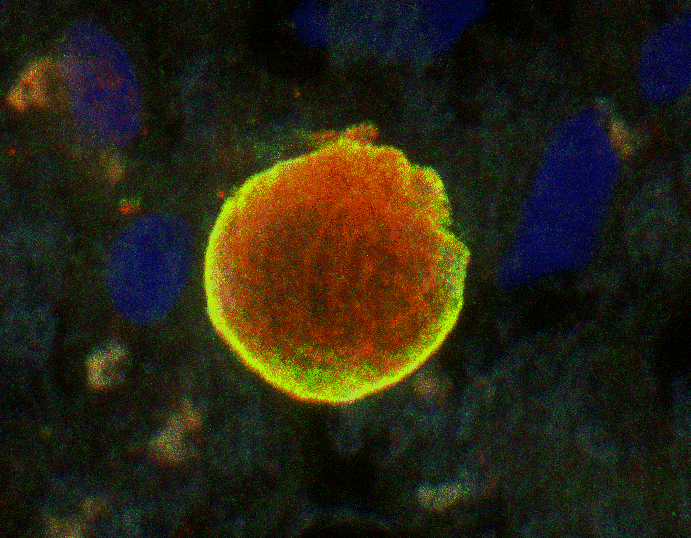

Supplement: Supplementary file 4 — Video V4 [file 41598_2018_31766_MOESM4_ESM.gif]
