# Supplementary material for: Exploring the elusive composition of corpora amylacea of human brain
Source: Sci Rep. 2018 Sep 10;8:13525. doi: 10.1038/s41598-018-31766-y (PMC6131176; doi:10.1038/s41598-018-31766-y)
Supplement: Supplementary file 5 — Figure S1 [file 41598_2018_31766_MOESM5_ESM.pdf]

Exploring the elusive composition of *corpora amylacea* of human brain

Elisabet Augé, Jordi Duran, Joan J. Guinovart, Carme Pelegrí, Jordi Vilaplana

Supplementary information, Figure S1

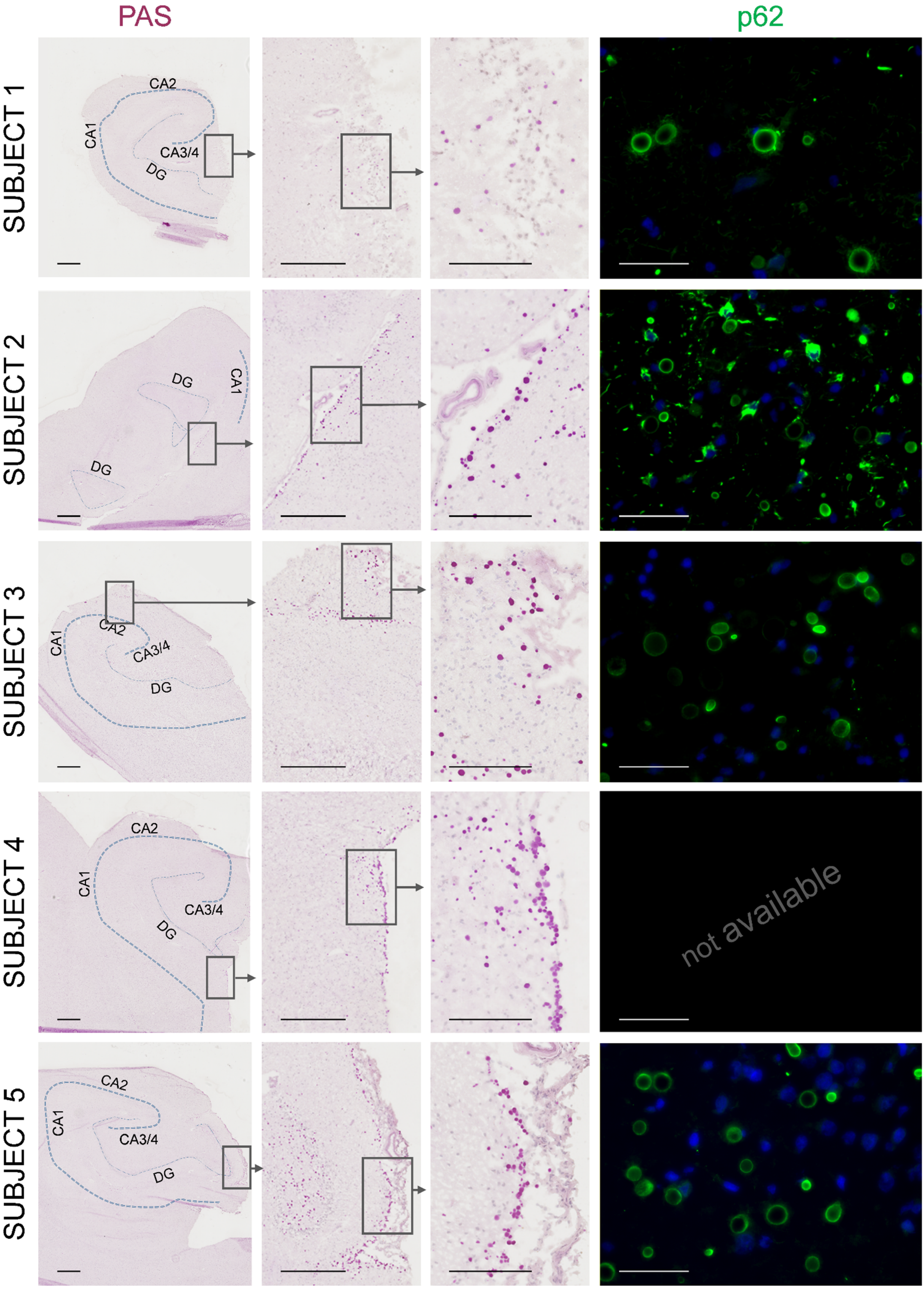
PAS
p62
SUBJECT 1
CA2
CA1
CA3/4
DG
SUBJECT 2
DG
CA1
DG
SUBJECT 3
CA1
CA2
CA3/4
DG
SUBJECT 4
CA2
CA1
CA3/4
DG
not available
SUBJECT 5
CA2
CA1
CA3/4
DG
